# A Case of Myoepithelial Hamartoma: Morphological Variation Supported by OCT4 Expression

**DOI:** 10.1155/2021/6617370

**Published:** 2021-02-26

**Authors:** Takehiro Tanaka, Kenji Nishida, Masaya Iwamuro, Satoru Kikuchi, Tadashi Yoshino

**Affiliations:** ^1^Department of Pathology, Okayama University, Graduate School of Medicine, Dentistry, and Pharmaceutical Science, Okayama, Japan; ^2^Department of Pathology, Okayama University Hospital, Okayama, Japan; ^3^Department of Gastroenterology and Hepatology, Okayama University, Graduate School of Medicine, Dentistry, and Pharmaceutical Science, Okayama, Japan; ^4^Department of Gastroenterological Surgery, Okayama University, Graduate School of Medicine, Dentistry, and Pharmaceutical Science, Okayama, Japan

## Abstract

In this report, we describe a patient with myoepithelial hamartoma, which is regarded as synonymous with adenomyosis and heterotopic pancreas. Endoscopy revealed a submucosal tumor in the antrum of the stomach. Subsequently, distal gastrectomy with Roux-en-Y reconstruction was performed. Histological findings of adenomyomatous lesion and heterotopic pancreatic tissue were observed in this lesion. The distribution of OCT4, which is a pluripotency marker, varied in each part.

## 1. Introduction

Submucosal tumors of the stomach are rare, with the exception of gastrointestinal stromal tumors and lymphomas. Myoepithelial hamartoma (MEH) was described in 5 cases for the first time by Magnus–Alsleben in 1903 [1]. MEHs can occur anywhere in the gastrointestinal tract, but they most commonly occur in the antrum of the stomach [2–5]. Histologically, an MEH is composed of hypertrophic smooth muscle bands surrounding diverse epithelial elements such as cystic glandular structures, pyloric glands, and pancreatic acini [6]. MEHs are regarded as synonymous with heterotopic pancreas [7, 8].

We report the case of a patient who presented with abdominal pain and MEH and discuss the association between adenomyoma and heterotopic pancreas.

## 2. Case Presentation

**Figure 1:**
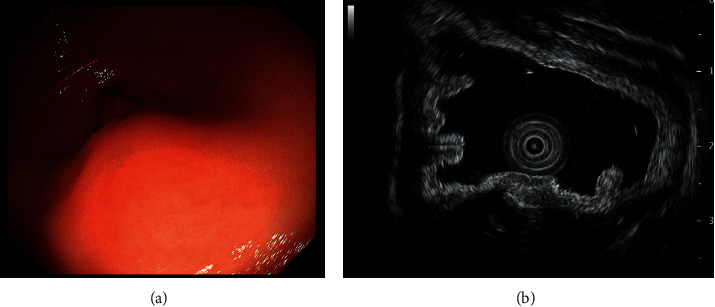
(a) Esophagogastroduodenoscopy (EGD) image. EGF revealed a pedunculated mass resembling a submucosal tumor in the antrum of the stomach. (b) Endoscopic ultrasonography image. The lesion was located in the submucosa and muscularis propria, and the mass was poorly defined and hypoechogenic.

A 39-year-old woman visited the previous hospital with a chief complaint of abdominal pain that lasted for a week. She had no history of gastrointestinal diseases. Subsequently, she underwent esophagogastroduodenoscopy, which revealed a submucosal tumor in the gastric antrum and a duodenal ulcer scar. Computed tomographic scanning showed wall thickening of the gastric antrum, after which the patient was referred to our hospital for further investigation and treatment. Esophagogastroduodenoscopy at our hospital revealed a pedunculated mass resembling a submucosal tumor in the antrum of the stomach (Figure 1(a)). Endoscopic ultrasonography revealed that the lesion was located in the submucosa and muscularis propria, and the mass was poorly defined and hypoechogenic (Figure 1(b)). Based on these findings, the presence of a gastrointestinal stromal tumor was suspected. Thus, an endoscopic ultrasonography-fine needle aspiration biopsy was performed for investigating the lesion. However, histological examination did not provide a diagnosis as the biopsy specimens revealed the presence of a small number of c-kit-negative spindle cells. Subsequently, distal gastrectomy with Roux-en-Y reconstruction was performed.

**Figure 2:**
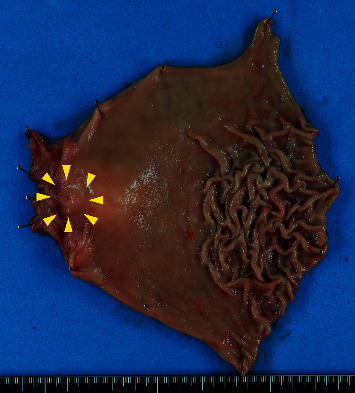
Macroscopic findings of the distal gastrectomy specimen. The lesion was located in the gastric antrum, and duodenum and was 3.7 cm long.

**Figure 3:**
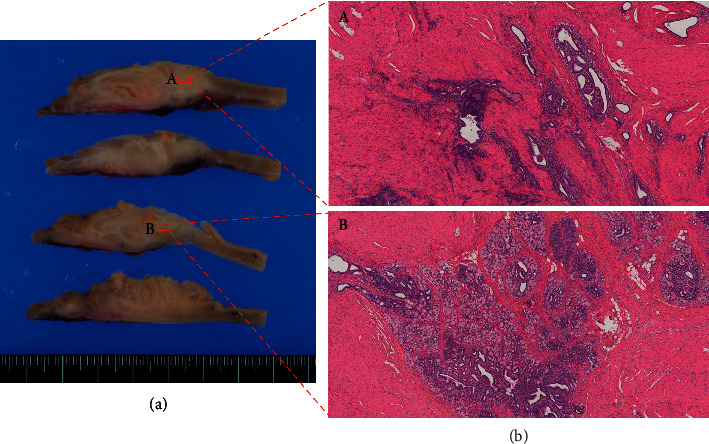
(a) Macroscopic findings. The resected specimen showed a vague nodule with dilated glands surrounded by edematous stroma in the submucosa and muscularis propria. (b) Histological findings. (A) Its appearance was similar to that of uterine adenomyosis. (B) The mass was composed of several types of glands, acini, and smooth muscle bundles.

**Figure 4:**
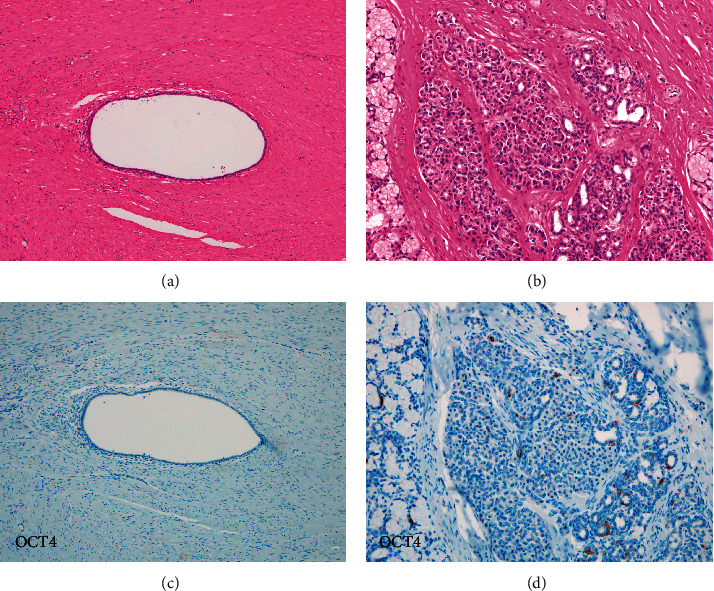
Histological findings. OCT 4 is not identified in the cells of the dilated glands ((a): HE staining; (b) OCT4). A small population of small gland cells was positive for OCT4 ((c): HE staining; (d): OCT4).

**Figure 5:**
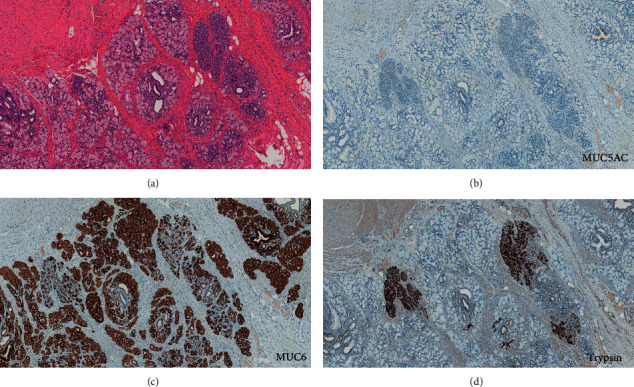
Histological findings. Organoid structure composed of large ducts surrounded by radially extending acini and small ducts resembling Brunner or pyloric glands (hematoxylin and eosin staining: HE (a)). Immunohistochemical study shows that MUC5AC is negative (b), whereas MUC6 is positive (c). Acinar cells are positive for trypsin (d).

The lesion was located in the gastric antrum and duodenum and was 3.7 cm long (Figure 2). The resected specimen showed a vague nodule with dilated glands surrounded by edematous stroma in the submucosa and muscularis propria (Figure 3(a)). Its appearance was similar to that of uterine adenomyosis (Figure 3(b)). Histologically, the mass was composed of several types of glands, acini, and smooth muscle bundles. Some ducts were simply dilated glands, but the remaining were more organoid, composed of large ducts surrounded by radially extending acini and small ducts resembling Brunner or pyloric glands (Figure 3(b)). In several areas, a transition from ductal epithelium to mucous gland epithelium was noted in the same structure. Lymphocytic infiltration was found around the dilated ducts, and abscess formation was also observed around a few ducts. The overlying mucosa showed reactive changes consisting of hypertrophic foveola and a mild atrophy of the pyloric glands. Furthermore, no cytological atypia, goblet cells, or pancreatic islets were identified. Immunohistochemically, the simple dilated glands' epithelium was positive for MUC6. OCT 4, which is a pluripotency marker, was not identified in the cells of the dilated glands (Figure 4). In the organoid pattern area, large ducts were negative for both MUC5AC and MUC6, and the surrounding small ducts were MUC6-positive but negative for MUC5AC (Figure 5). Furthermore, acinar cells tested positive for trypsin (Figure 4). A small population of small gland cells was positive for OCT4 (Figure 5). Test for INSM-1 did not outline aggregates of endocrine cells.

## 3. Discussion

We reported the presence of an MEH with various histological features. MEH is synonymous with adenomyosis and ectopic pancreas. In this case, there were two histological components: “adenomyosis,” in which only the ductal structure that expanded to the smooth muscle bundle was seen and “heterotopic pancreas,” ducts, and acini. Although different names have been given due to the differences in histological findings, we essentially have the same two types of lesions; both components appear in the same lesion in this case.

In this study, OCT4-positive cells were not found in the adenomyosis part; they were found in the part showing a more organoid structure. OCT4 is a pluripotency marker [9–11], suggesting that pluripotent cells may be required for the differentiation into pancreatic tissue. This result suggests that the presence or absence of pluripotent cells results in differences in tissue organization.

There is a theory that heterotopic pancreas is a stray pancreatic tissue during embryonic development [12–14]; however, in some cases such as adenomyosis where the pancreatic tissue is not identified, it is difficult to explain its presence. On the contrary, the findings of acinar metaplasia of gastric mucosa are occasionally observed in type A gastritis, where the pluripotent cells in the stomach are differentiated to various degrees, and the result of hyperplasia is the essence of the lesion. Thus, we believe that the name “myoepithelial hamartoma” is appropriate [15].

Neoplastic lesions arising from ectopic pancreatic tissue have been reported [16, 17], most of which are adenocarcinomas similar to pancreatic ductal carcinoma; however, there are also reports of acinar cell carcinomas [18] and neuroendocrine tumors [19].

Mucosal incision-assisted biopsy (MIAB) is powerful method for the diagnosis of subepithelial lesions [20, 21]; we might have tried MIAB after EUS-FNA failure, but the patient's symptom got worse, and hence, we decided to have surgery. The lesion was located from the antrum of stomach to the bulbus of duodenum across the pylorus, and distal gastrectomy was selected for the treatment instead of laparoscopic and endoscopic cooperative surgery (LECS) [22].

To our knowledge, no studies have reported the distribution of OCT4 in such tumors; however, analysis of cases is needed to explain the tumor genesis. Since there are many mysteries about the development of MEH, further examination is needed with regard to it.

## References

[1] Magnus-Alsleben E. (1903). Adenomyome des pylorus. *Virchows Archiv für Pathologische Anatomie und Physiologie und für Klinische Medizin*.

[2] Ikegami R., Watanabe Y., Tainaka T. (2005). Myoepithelial hamartoma causing small-bowel intussusception: a case report and literature review. *Pediatric Surgery International*.

[3] Trifan A., Târcoveanu E., Danciu M., Huţanaşu C., Cojocariu C., Stanciu C. (2012). Gastric heterotopic pancreas: an unusual case and review of the literature. *Journal of Gastrointestinal and Liver Diseases: JGLD*.

[4] Zarling E. J. (1981). Gastric adenomyoma with coincidental pancreatic rest: a case report. *Gastrointestinal Endoscopy*.

[5] Ryan A., Lafnitzegger J. R., Lin D. H., Jakate S., Staren E. D. (1998). Myoepithelial hamartoma of the duodenal wall. *Virchows Archiv*.

[6] Vandelli A., Cariani G., Bonora G., Padovani F., Saragoni L., Dell’Amore D. (1993). Adenomyoma of the stomach. *Surgical Endoscopy*.

[7] Teresa Rosanna P., Francesco M., Salvatore V. (2007). Myoepithelial hamartoma of the stomach simulating a gastric carcinoma: a case report. *Tumori*.

[8] Babal P., Zaviačič M., Danihel L. (1998). Evidence that adenomyoma of the duodenum is ectopic pancreas. *Histopathology*.

[9] Medvedev S. P., Shevchenko A. I., Mazurok N. A., Zakiian S. M. (2008). OCT4 and NANOG are the key genes in the system of pluripotency maintenance in mammalian cells. *Genetika*.

[10] Wang Y., Lanzoni G., Carpino G. (2013). Biliary tree stem cells, precursors to pancreatic committed progenitors: evidence for possible life-long pancreatic organogenesis. *Stem Cells*.

[11] Stefanovic S., Abboud N., Désilets S., Nury D., Cowan C., Pucéat M. (2009). Interplay of Oct4 with Sox2 and Sox17: a molecular switch from stem cell pluripotency to specifying a cardiac fate. *Journal of Cell Biology*.

[12] Yamagiwa H., Onishi N., Nishii M. (1992). Heterotopic pancreas of the stomach. *Pathology International*.

[13] Terada T. (2010). Heterotopic pancreatic tissue of the stomach: report of three cases and consideration of its histogenesis. *Case Reports in Gastroenterology*.

[14] Filip R., Walczak E., Huk J. (2014). Heterotopic pancreatic tissue in the gastric cardia: a case report and literature review. *World Journal of Gastroenterology*.

[15] Takahashi Y., Fukusato T. (2011). Adenomyoma of the small intestine. *World Journal of Gastrointestinal Pathophysiology*.

[16] Chapple C. R., Muller S., Newman J. (1988). Gastric adenocarcinoma associated with adenomyoma of the stomach. *Postgraduate Medical Journal*.

[17] Song D. E., Kwon Y., Kim K.-R., Oh S. T., Kim J.-S. (2004). Adenocarcinoma arising in gastric heterotopic pancreas: a case report. *Journal of Korean Medical Science*.

[18] Sun Y., Wasserman P. G. (2004). Acinar cell carcinoma arising in the stomach: a case report with literature review. *Human Pathology*.

[19] Tanaka T., Omote R., Okazaki N., Yanai H., Yoshino T. (2018). Gastric neuroendocrine tumor arising from heterotopic pancreas. *Clinical Journal of Gastroenterology*.

[20] Osoegawa T., Minoda Y., Ihara E. (2019). Mucosal incision‐assisted biopsy versus endoscopic ultrasound‐guided fine‐needle aspiration with a rapid on‐site evaluation for gastric subepithelial lesions: a randomized cross‐over study. *Digestive Endoscopy*.

[21] Minoda Y., Chinen T., Osoegawa T. (2020). Superiority of mucosal incision-assisted biopsy over ultrasound-guided fine needle aspiration biopsy in diagnosing small gastric subepithelial lesions: a propensity score matching analysis. *BMC Gastroenterology*.

[22] Matsuda T., Nunobe S., Kosuga T. (2017). Laparoscopic and luminal endoscopic cooperative surgery can be a standard treatment for submucosal tumors of the stomach: a retrospective multicenter study. *Endoscopy*.

